# Book Review: Memory and the Self: Phenomenology, Science and Autobiography

**DOI:** 10.3389/fpsyg.2018.00177

**Published:** 2018-02-16

**Authors:** Kourken Michaelian

**Affiliations:** Department of Philosophy, University of Otago, Dunedin, New Zealand

**Keywords:** memory, self, episodic memory, philosophy of memory, embodied cognition

The primary aim of this ambitious book is to argue for the existence of a hitherto-overlooked form of memory. “Rilkean memory” (so called after the poet Rilke) is, according to Rowlands, “a type of involuntary autobiographical memory that is not Freudian, neither implicit nor explicit, neither procedural nor declarative and neither episodic nor semantic” (67). Chapter 1 begins by introducing the topic of the presence of self in episodic memory. Distinguishing between the act of remembering and the remembered content, Rowlands suggests that the act of remembering may sometimes outlive the remembered content, persisting in a “new, mutated form” (12); this is Rilkean memory. Ultimately, he will argue, the persistence of one's memories in this form compensates for the limitations of the content provided by episodic memory and thus plays an important role in underwriting the continuity of the self.

Chapter 2 focuses on episodic memory, the specificity of which, Rowlands suggests, lies not in the kind of content it provides but rather in the way in which it provides content: an episodic memory includes not only an act of remembering and a remembered episode but also a specific mode of presentation of the episode. While the mark of episodicity is an important open problem (Perrin and Rousset, 2014), it is not obvious that this invocation of the Fregean notion of a mode of presentation sheds much new light on it. It may or may not be right to say that, in episodic memory, “you must remember the episode *as* one that you formerly experienced” (49), but, either way, it is not clear what this might add to definitions of episodicity in terms of autonoesis advanced by Tulving (1985), Klein (2015), and others.

Since Rilkean memories are not contentful, they (by definition) do not involve modes of presentation, and the notion of a mode of presentation therefore likewise seems to shed little light on the relationship between episodic memory and Rilkean memory or on the nature of the latter. Chapter 3 contains the book's most extended discussion of Rilkean memory, which is said to come in both embodied and affective varieties. Both varieties qualify as autobiographical memory, for Rowlands, in virtue of the fact that they come about when “the act of [episodic] remembering becomes divorced from what is remembered” (73). One of his central examples of embodied Rilkean memory is that of a runner whose odd gait developed as a means of avoiding pain when running with knee problems; the claim is that the disposition to run with this gait may qualify as a form of memory even if the runner no longer remembers the pain and that the disposition could only have come about via earlier episodic memories of the pain. But while the modification of behavioral dispositions at issue here amounts to learning and therefore trivially involves memory, episodic memory, in particular, would appear to play no necessary role, as the subject's behavioral dispositions might be modified simply through conditioning. Even if, as Rowlands suggests, a form of short-term declarative memory necessarily mediates between the runner's painful experiences and the modification of his behavioral dispositions, this would not appear to be episodic memory, which, as Rowlands himself emphasizes elsewhere in the book, is a form of long-term memory resulting from an extended consolidation and reconsolidation process. Moreover, even if (short-term) episodic memory does play a mediating role here, it would seem to be misleading at best to claim that Rilkean memories emerge from episodic memories when “the act of remembering becomes divorced from what is remembered,” for the activation of the runner's behavioral dispositions is an act of a fundamentally different kind than the act—involving the retrieval or reconstruction of content—involved in episodic remembering. Ultimately, the path leading from specific acts of episodic remembering (in which the self-figures through the episodic mode of presentation) to the development of either behavioral or affective dispositions of the relevant sort (in which the self-figures only in a looser sense) remains somewhat difficult to grasp.

Chapter 4 turns to the autobiographical self, which Rowlands defines as whatever one's autobiography would be about. The following two chapters interrogate the ability of memory to “make us who we are,” given that we are prone to both forgetting (Chapter 5) and error (Chapter 6). The thought is that the autobiographical self, if it corresponds to the object of an autobiography, corresponds to the object of an autobiography that has been heavily redacted and rewritten. Rowlands then argues, in Chapter 7, that, just as, in a written narrative, unity of style can make up for gaps in content, Rilkean memories can provide one's life with a sort of unity sufficient to make up for the gaps in content introduced by forgetting and error. The analogy between Rilkean memory and literary style is, however, somewhat puzzling. In the case of a written narrative, style can weld gappy contents together into a coherent whole because it is a matter of how content is presented. In the case of the autobiographical self, though Rilkean memory may ensure a degree of behavioral and affective similarity across time, it is, given that it is not a matter of how content is presented, difficult to see how it might make up for the gappiness of the contents provided by episodic memory.

Chapter 8 further discusses the presence of self in memory, arguing that it is a necessary condition for episodicity. Chapter 9 provides a more detailed discussion of the content of memory, arguing that the remembered episode is not the same as the remembered content. Rowlands opts here for a form of direct realism, maintaining that “[i]n remembering content, I am in direct contact with the episode” (188); it would have been interesting to see some discussion of how his version of the view handles problems for other forms of direct realism posed by phenomena such as confabulation. Chapter 10, finally, briefly ties together the various threads of the book's argument.

Rowlands' book is a major contribution to the philosophy of memory. It not only proposes a novel interpretation of familiar psychological findings by means of an original application of relevant philosophical concepts but also proposes an entirely unprecedented concept, that of Rilkean memory. While his argument for the existence of Rilkean memory, its relationship to episodic memory, and its role in ensuring the unity of the self may leave some readers unconvinced, Rowlands has done an admirable job of making a case for a novel view, and those who wish to oppose the view will have their work cut out for them. We should look forward to more work from the author on the same topic.

## Author contributions

The author confirms being the sole contributor of this work and approved it for publication.

### Conflict of interest statement

The author declares that the research was conducted in the absence of any commercial or financial relationships that could be construed as a potential conflict of interest.
